# Coconut Testa Flour Sub-Fractions: Correlation Between FTIR Spectral Data and α-Glucosidase Inhibitory Activities

**DOI:** 10.3390/foods13213418

**Published:** 2024-10-27

**Authors:** Rasika Gunarathne, Savani Ulpathakumbura, Nazrim Marikkar, Lalith Jayasinghe, Jun Lu

**Affiliations:** 1 Auckland Bioengineering Institute, University of Auckland, Auckland 1142, New Zealand; kgun686@aucklanduni.ac.nz; 2 National Institute of Fundamental Studies, Hanthana Road, Kandy 20000, Sri Lanka; buthsara.ul@nifs.ac.lk (S.U.); lalith.ja@nifs.ac.lk (L.J.); 3 Maurice Wilkins Centre for Biodiscovery, Auckland 1142, New Zealand; 4 Department of Food and Agriculture Technology, Yangtze Delta Region Institute of Tsinghua University, Jiaxing 314006, China

**Keywords:** coconut testa flour, crude extracts, FTIR, Alpha-glu inhibition, PLS, OPLS

## Abstract

Fourier-transform infrared spectroscopy (FTIR) serves as a rapid analytical technique to characterize food specimens chemically. The purpose of this study was to investigate the potential of FTIR combined with multivariate statistics to detect Alpha-glucosidase (Alpha-glu) inhibitory activities of a non-cereal flour-like coconut testa flour (CTF). CTF of five distinct local cultivars was sequentially extracted with hexane, ethyl acetate (EtOAc), and methanol (MeOH) to assay the Alpha-glu inhibitory activity. FTIR spectra of CTF extracts were obtained within the range of 4000–500 cm^−1^ and the prominent spectral peaks obtained for both hexane and EtOAc extracts were roughly similar but some additional peaks were observed in EtOAc extracts representing phenolic constituents. The major absorbance peaks found in MeOH extracts were primarily indicative of the occurrence of the hydroxyl group associated with carbohydrates and phenolic compounds. The multivariate predictive models developed using partial least squares (PLS) and orthogonal partial least squares (OPLS) regression analyses indicated a strong correlation between Alpha-glu inhibitory activity and spectral data. Models developed for the spectral regions 3700–2800 cm^−1^ and 1800–500 cm^−1^ exerted the highest regression coefficients with the lowest root mean square errors. In OPLS regression analysis, the model obtained with third-derivative spectral data was identified as the best, exhibiting the highest regression coefficients and the lowest root mean square errors. Both PLS and OPLS regression analyses indicated a potential correlation of Alpha-glu inhibitory activity with FTIR spectral regions. Notably, OPLS models offered enhanced interpretability of the model parameters. This study suggests that the application of multivariate regression analysis of FTIR spectral data on coconut-based products could help to detect Alpha-glu inhibitory activities.

## 1. Introduction

Diabetes mellitus is a major endocrine disorder that arises due to the absolute or relative deficiency of insulin [1]. The high prevalence of diabetes and the steady increment of counts all over the world have become a burden to the health sector. Type II diabetes is considered to be more prevalent than Type I, and is generally characterized by postprandial hyperglycemia [2]. The digestive enzymes present in the human small intestine play a pivotal role in regulating postprandial blood glucose levels [3]. Therefore, inhibiting the Alpha-glu enzyme to retard glucose absorption represents an effective therapeutic approach for managing postprandial hyperglycemia [4].

The anti-hyperglycemic effects of various plant-based foods have been explored through both in vitro and in vivo assays, focusing on carbohydrate-hydrolyzing enzymes, namely, α-glucosidase and α-amylase. In vitro investigations primarily involve enzymatic assays associated with glucose metabolism. Further, these investigations involve the assessment of the anti-diabetic effectiveness of biological entities, drugs, or bioactive molecules by evaluating biochemical indicators such as insulin levels, fasting blood glucose, and serum protein concentrations [5]. As these in vitro methods are time-consuming and require multiple chemical reagents, there is an urgency to discover rapid analytical methods to detect the Alpha-glu inhibitory activity of food substances [6].

With the recent advancement in electronics and computer technology, FTIR spectroscopy has widened its scope as a time-saving, convenient analytical instrument for the chemical mapping of various agricultural products [7,8]. One of the most significant advantages of this method is its requirement for minimal reagents. Several previous reports have shown that FTIR spectral data coupled with multivariate statistics would be effective in developing predictive models for the detection of Alpha-glu inhibition in some plant materials [6,9,10]. In this study, we attempted to explore the correlations between Alpha-glu inhibitory activity and FTIR data of CTF of different cultivars. The outcomes of this study will not only contribute to fulfilling the need for a rapid method for exploring Alpha-glu enzyme inhibitory activity but will also provide insights for further studies on this aspect.

## 2. Methodology

### 2.1. Materials

**Figure 1:**
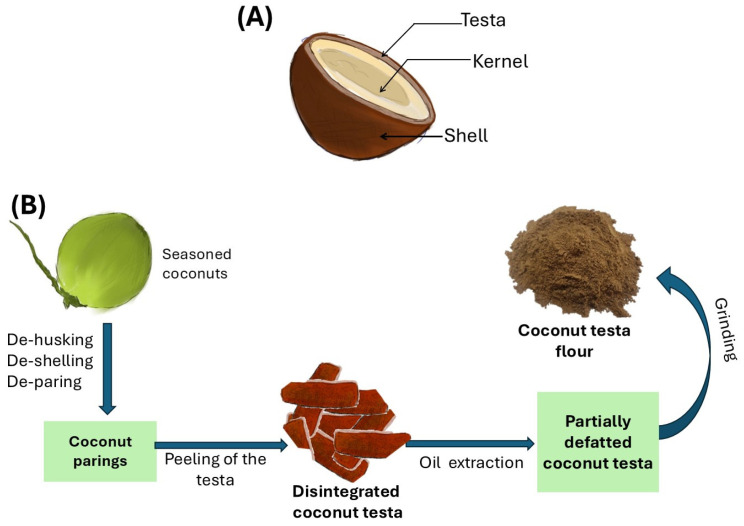
A summary of preparation of CTF: (**A**) parts of a coconut; (**B**) steps of preparation of CTF.

Coconuts of twelve-month maturity of five local cultivars (COM, RT, SR, GT, and TxT) were obtained from varietal blocks at the Coconut Research Institute, Lunuwila, Sri Lanka. A total of fifty coconuts from each cultivar were randomly selected, followed by seasoning and de-husking. The coconut testa flour (CTF) was prepared following the method presented by Marasinghe et al. [11]. Briefly, mature coconuts underwent de-husking, de-shelling, and de-paring. The testa, separated from the kernel, was disintegrated and dried (70 °C for 8 h) in a cabinet-type dehydrator (Wessberg, Martin, Germany), followed by removing the oil by cold-press oil extraction using a micro-oil expeller (Komet DD85 machine, Mönchengladbach, Germany). The remaining residues (<15% oil) were ground in a general-purpose grinder until fine powder was obtained to prepare CTF and stored in refrigerated (4 °C) conditions for further analysis. Unless otherwise stated, all chemicals in this research study were of analytical grade. A summary of the preparation of CTF is illustrated in Figure 1.

### 2.2. Sub-Fraction Preparation

Sub-fraction preparation of the CTF was performed according to the method described by Gunarathne et al. [7]. A 250 g portion of CTF from individual cultivars was sequentially extracted using non-polar, mid-polar, and polar solvents. Therefore, each extraction was performed for 30 min with 1000 mL of hexane (≥95%), EtOAc (≥99.5%), and MeOH (≥99.8%) in a sonicating machine (Rocker ultrasonic cleaner, model—Soner 206) operating at 50 KHz. In each case, the extraction process was replicated three times consecutively for equal time intervals. The solvent extracts of individual cultivars were concentrated by employing a rotary evaporator under low pressure and at a temperature of 50 °C for 10–15 min (Heidolph, Laborota 4000). The semi-solid extracts were freeze-dried using a bench-top pro-freeze dryer (ESCO, model—FDL-2S8, Singapore) at −30 °C (1 h), −25 °C for 16 h under a pressure level of 0.005 mbar. The crude extracts of individual cultivars were kept at −18 °C until further analysis.

### 2.3. Alpha-glu Inhibitory Assay

Assessment of the inhibitory activity of CTF sub-fractions against Alpha-glu was performed by following the method described by Gunarathne et al. [7]. Briefly, a range of concentrations of the crude extracts (3.91–1000 ppm) was prepared in 3% DMSO-added distilled water. Following that, 100 µL of 30 mM phosphate buffer (pH 6.5) was introduced into 96-well microplates and, subsequently, combined with 25 µL of the sample solution. Thereafter, 25 µL of Alpha-glu enzyme (12.5 µL/mL) was introduced and the mixture was incubated at 37 °C for 5 min. Following this, 50 µL of *p*-nitrophenyl-α-D-glucopyranoside (0.8 mg/mL) was introduced to the mixture and incubated for another 30 min at 37 °C. In this study, the positive control utilized was acarbose (Glucobay tablet). The absorbance value was quantified at 410 nm and the percentage of α-Alpha-glu inhibitory activity was computed using the following equation. The IC_50_ values were determined by creating a plot that depicted the percentage of Alpha-glu inhibition against the sample concentration of each extract.

$$\mathrm{Percentage}\;\mathrm{Inhibition}\;\mathrm{of}\;\mathrm{Alpha}\text{-}\mathrm{glu}\;\mathrm{activity}=\frac{\delta A_{\mathrm{control}}\;-\;\delta A_{\mathrm{sample}}}{\delta A_{\mathrm{control}}}\times 100$$

where δA_control_ = absorbance_control_ − absorbance_control blank_; δA_sample_ = absorbance_sample_ − absorbance_sample blank_.

### 2.4. FTIR Measurements

Measurements were taken employing an FTIR Nicolet iS50 spectrometer (Thermo Nicolet, Madison, WI, USA) equipped with deuterated triglycine sulfate, a KBr detector, and KBr beam splitter. The spectra were recorded using the KBr pallet method in the region of 4000–500 cm^−1^ by co-adding at 64 scans, with a resolution of 8 cm^−1^, as explained before by Mittal et al. [12]. The individual sample was blended with FTIR-grade KBr (≥99% trace metals basis, Sigma Aldrich, St. Louis, MO, USA) at a 1:90 ratio and then compressed into a pallet. At each data point, spectra were recorded as absorbance values in 4 replicates.

### 2.5. Statistical Analysis

Alpha-glu inhibitory measurements were acquired in triplicate data (*n* = 3) and the results were reported as mean ± standard deviation (SD). The data were subjected to statistical analysis using one-way ANOVA with the Minitab 17 software package. In cases where the F values were found to be significant, the mean differences were calculated using Tukey’s test at the 95% significance level.

### 2.6. Spectral Preprocessing

Spectral preprocessing of raw spectra was performed using the manufacturer’s software, OMNIC operating system, version 7.0, Thermo Nicolet. Herein, the raw spectra underwent baseline correction and scale normalization. The final spectra obtained were then utilized to identify the prominent spectral bands present in the range of 4000–700 cm^−1^.

### 2.7. PLS Regression Analysis

PLS regression analysis of spectral data was performed employing the Unscrambler 9.7 (Camo, Beavercreek, OH, USA) software, adopting the procedure outlined by Gunarathne et al. [8]. Briefly, data from a total of 15 crude extracts were collected with 4 replicates each. These were divided randomly into two sets: one with 40 elements for calibration and cross-validation and the other with 20 elements for predictive models. The total spectral range (3700–700 cm^−1^) and different sub-spectral ranges (A: 3700–2800 cm^−1^; B: 1800–1700 cm^−1^; C: 1700–1500 cm^−1^; D: 1500–900 cm^−1^; E: 900–500 cm^−1^) were used in developing predictive models. The model parameters—the coefficient of determination of calibration (R_c_^2^), coefficient of determination of prediction (R_p_^2^), coefficient of determination of cross-validation (Rcv^2^), root mean square errors of calibration (RMSEC), root mean square errors of prediction (RMSEP), and root mean square errors of cross-validation (RMSECV)—were compared to identify the best predictive models for Alpha-glu inhibition.

### 2.8. OPLS Regression Analysis

OPLS regression analysis of spectral data of CTF sub-fractions was carried out using the SIMCA 14.0 version (Umetrics, Umeå, Sweden) in accordance with the protocol stated by Easmin et al. [6]. The spectral regions that produced the best PLS model were selected for the OPLS analysis. A total of 60 independent observations were used to develop and validate the models. The model parameters—R^2^Y, regression coefficient, Q^2^, predictive regression coefficient, RMSEE, root mean square error of estimation, and RMSE_CV_, root mean square error of cross-validation—were compared to identify the best predictive models for Alpha-glu inhibition.

## 3. Results

### 3.1. Alpha-glu Inhibitory Activity

The Alpha-glu inhibitory activities of the CTF sub-fractions are shown in Table 1. All tested sub-fractions of CTF exhibited inhibitory activity against the Alpha-glu enzyme; the inhibitory activities were found to vary based on the cultivar differences as well as the type of solvent used for extraction. Further, the inhibitory activities of the different sub-fractions were concentration-dependent; as such, the activities tended to show gradual increments with increasing concentrations. Nevertheless, they become steady after reaching the maximum percentage inhibition.

IC_50_ values of the hexane extracts ranged from 8.38 ± 0.52 ppm to 65.91 ± 3.92 ppm. Among these extracts, GT provided the lowest IC_50_ value, while TT resulted in the highest IC_50_ value. Notably, the differences in IC_50_ values were statistically significant (*p* < 0.05), except for the SR and TT cultivars. In EtOAc extracts, the TT cultivar resulted in the lowest IC_50_ value (7.82 ± 0.40 ppm), while COM showed the highest IC_50_ value (56.64 ± 3.37 ppm). However, results corresponding to only COM cultivars were significantly (*p* < 0.05) different from the rest. As for the MeOH extracts, IC_50_ values ranged from 22.53 ± 0.26 ppm to 403.32 ± 17.24 ppm, with the highest value recorded for the COM cultivar and the lowest for the GT cultivar. As mentioned before, only COM showed an IC_50_ value that was significantly (*p* < 0.05) different from the values of the other cultivars.

### 3.2. FTIR Spectral Characterization

The FTIR chemical mapping of the three sub-fractions is summarized as shown in Table 2. The spectral bands that appeared in individual extracts were assigned to their corresponding functional groups. Briefly, the FTIR spectra of hexane and EtOAc extracts showed almost similar contours except for a few spectral bands. The spectral bands of these extracts indicated the presence of lipid molecules, which was explained by the dominant spectral band of the ester bond of triacylglycerols. Meanwhile, the additional peaks found in EtOAc extracts indicated the presence of phenolic constituents. When compared to hexane and EtOAc, the spectra of MeOH extracts showed drastic variations, indicating notable differences in their chemical composition. The prominent peak in MeOH spectra that appeared due to the stretching vibrations of hydroxyl groups could be associated with carbohydrates and phenolic compounds. Moreover, the minor peaks found in these spectra confirmed the existence of protein and lipids in MeOH extracts. The observed changes in the chemical composition of the extracts could be a direct result of the sequential extraction as it enhances the polarity-based separation of chemical constituents.

### 3.3. Predictive Models

**Figure 2:**
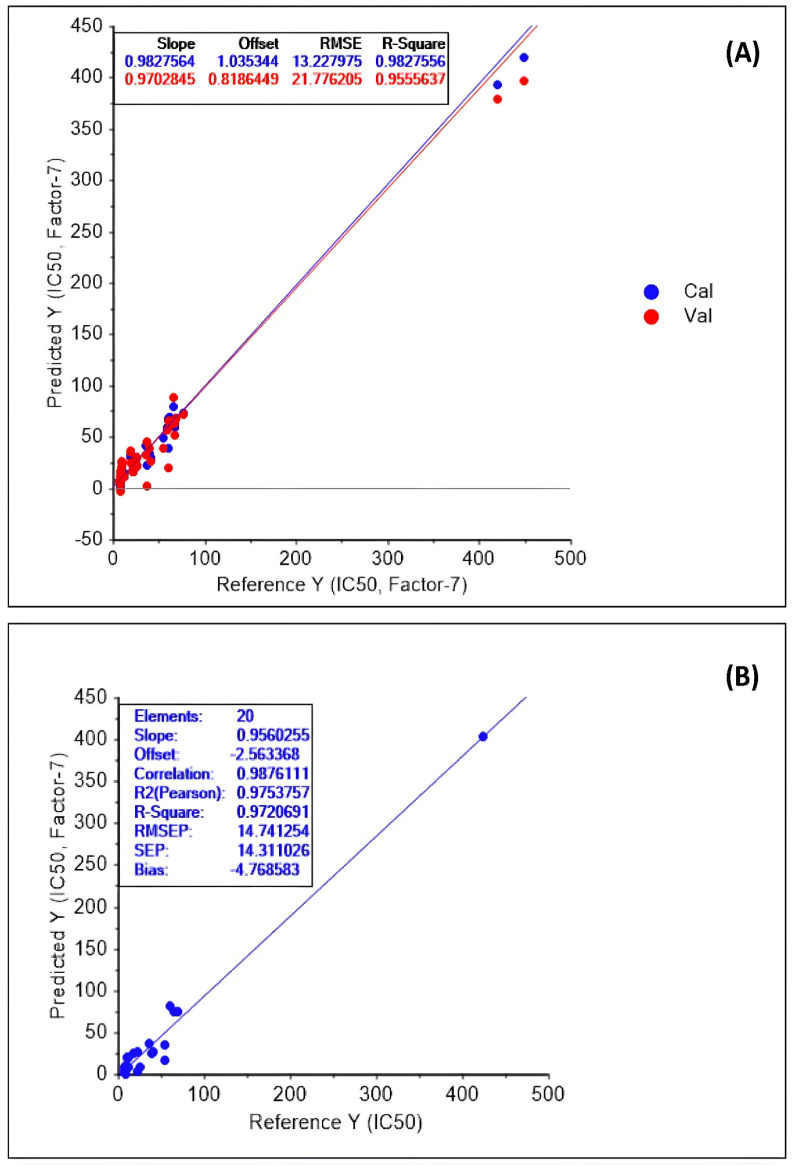
PLS regression plots of Model 12: (**A**) regression lines of calibration and validation; (**B**) regression line of prediction.

The predictive models based on the PLS regression analysis of spectral results and inhibitory activity of Alpha-glu of the CTF sub-fractions are shown in Table 3. Rc^2^, Rcv^2^, and Rp^2^ of the predictive models obtained for spectral regions, namely A, B, C, D, and E, showed high correlations that were more or less close to 1. Among these predictive models, “Model 5” was found to have the greatest Rc^2^ (0.96), Rcv^2^ (0.93), Rp^2^ (0.98), and the least RMSEC (19.34), RMSECV (26.58), and RMSEP (13.91). However, “Model 2” resulted in the smallest Rc^2^ (0.59), Rcv^2^ (0.33), Rp^2^ (0.50) and the greatest RMSEC (64.83), RMSECV (84.50), and RMSEP (62.81), exhibiting its low precision. Among the predictive models developed employing multiple regions, “Model 12” had the greatest Rc^2^ (0.98), Rcv^2^ (0.96), and Rp^2^ (0.97), resulting in the lowest RMSEC (13.23), RMSECV (21.78), and RMSEP (14.74). The calibration and validation plots based on “Model 12” are given in Figure 2A, while the corresponding prediction plot is given in Figure 2B. Past studies, including Gunarathne et al. [8], showed that engaging multiple spectral regions in developing predictive models could be more useful in improving precision. It would become further evident that the chemical constituents representing the spectral ranges, namely A, C, D, and E, have had a strong impact, while the constituents representing region B had minimal influence on the inhibitory activity of Alpha-glu. As such, the spectral zones A, C, D, and E, representing the phenolic and alcoholic constituents (Table 2), suggest potent Alpha-glu enzyme inhibitory activity, while the fatty molecules representing region B might not exert strong inhibition towards Alpha-glu.

### 3.4. OPLS Modeling

**Figure 3:**
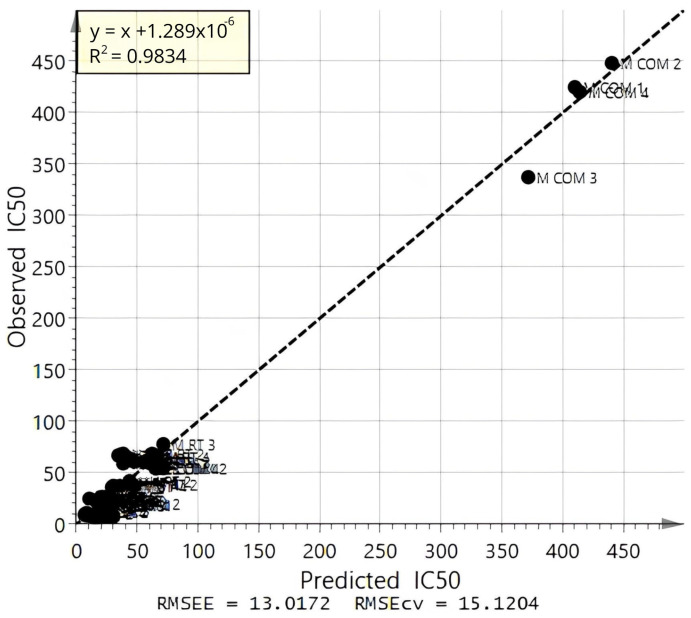
The regression line between the predicted and observed IC_50_ values.

The results of the OPLS analysis of Alpha-glu inhibitory activity and the FTIR spectra are shown in Table 4. The values of the regression coefficient (R^2^Y), predictive regression coefficient (Q^2^), root mean square error of estimation (RMSEE), and root mean square error of cross-validation (RMSECV) obtained for the different data filters were compared. According to Table 4, the model developed with third derivatives of spectral data (Model IV) showed the highest R^2^Y (0.983) and Q^2^ (0.976) values as well as the lowest values for RMSEE (13.017) and RMSECV (15.120). The model developed with second-derivative spectral data was the second-best model as it showed the second largest R^2^Y (0.980) and Q^2^ (0.969) values and second lowest RMSEE (14.246) and RMSECV (16.936) values. The first-derivative spectra, MSC spectra, and SNV spectra also showed high R^2^Y (>0.975) and Q^2^ (>0.960) and considerably low RMSEE (<16.301) and RMSECV (<19.371) when compared with the model obtained for normal spectra, which possessed the lowest R^2^Y and Q^2^ values and the highest RMSEE and RMSECV values. The regression line between the observed and predicted IC_50_ values of Alpha-glu inhibitory activity of “Model IV” is depicted in Figure 3. It exhibits a high R^2^Y value of 0.983, which explains the high predictive power and the good fit of the model.

**Figure 4:**
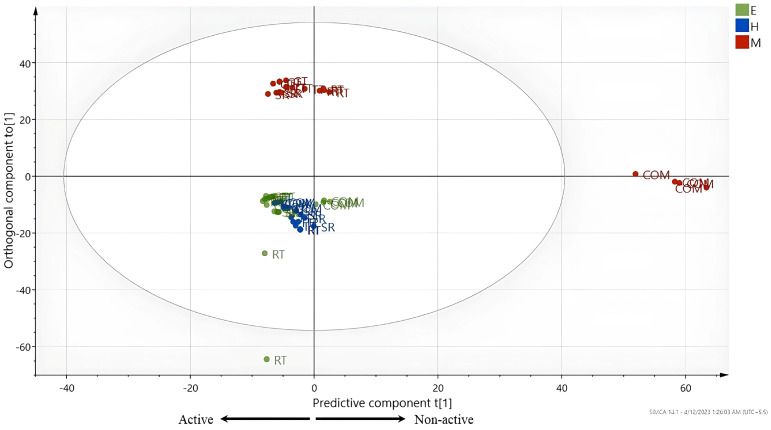
Orthogonal partial least squares score scatter plot of crude extracts of coconut testa flour.

Figure 4 depicts the OPLS score scatter plot of CTF sub-fractions. The MeOH extract of the COM cultivar is distinctly separated from the other extracts along the predictive component, which is positioned on the positive side of the plot. The MeOH extract of the RT cultivar and EtOAc extract of the COM cultivar were separated into the positive semicircle, while the other extracts remained in the negative semicircle. There was a remarkable separation between the MeOH extract of the COM cultivar and other crude extracts because of the lowest activity displayed by the MeOH extract of the COM cultivar.

## 4. Discussion

The inhibitory activities were found to vary based on both cultivar differences and the solvent type used for extraction. The amount and the type of specific constituents responsible for Alpha-glu inhibitory activity in coconut testa may differ depending on the cultivar. Further, since sequential extraction was employed during the preparation of the crude extracts, the type of compounds in each solvent extract varied according to their polarity. Non-polar, mid-polar, and polar constituents responsible for Alpha-glu inhibitory activity may concentrated into hexane, ethyl acetate, and methanol extracts of each cultivar, respectively. Thus, the variations in inhibitory activities can be attributed to the cumulative effects of cultivar differences as well as the type of solvent used for extraction.

According to Gunarathne et al. [7], the CTF of all local coconut cultivars was abundant in phenolics and flavonoids, which could act as potent substrates to exert anti-hyperglycemic and antioxidant activities. This is in concurrence with the results of Adekola et al. [13], who reported on the anti-hyperglycemic properties of coconut testa of Malaysian origin. As reported previously, phytochemicals present in CT such as phenols, flavonoids, tannin, glycosides, alkaloids (quinines), coumarin, resin, terpenes, saponin (amphipathic glycosides), fats, and oil may act as potent Alpha-glu inhibitors [7,14,15]. The phenols and flavonoids found in the MeOH extracts of CTF were reported to have a strong correlation to its Alpha-glu inhibitory activity [7]. Overall, results suggest that the non-polar compounds present in CTF may play a crucial role in the inhibition of this enzyme. According to a previous study, constituents like 1,3-Dipalmitolein and cis-9-octadecenoic were responsible for the potent Alpha-glu inhibitory activity displayed by hexane extracts of sea cucumber [16]. Another study reported that non-polar constituents found in moringa extracts, such as sterols (campesterol, β-sitosterol, and stigmasterol) and terpenoids, might be responsible for the Alpha-glu inhibitory activity [17]. Moreover, some terpenoids found in Chaga mushrooms were also reported to possess inhibitory activity against Alpha-glu [18].

Development of rapid methods to detect the Alpha-glu enzyme inhibitory activity of plant materials has increasingly become the focus of many studies. They emphasize that FTIR combined with multivariate data analysis could be a successful approach for this purpose. Particularly, PLS regression and OPLS regression analyses were employed for the development of statistical models. Further, the results of the multivariate analysis were able to highlight the functional moieties or metabolites that are responsible for anti-glucosidase activity [6,9,10]. In fact, OPLS is a modified version of PLS, which has the ability to process a large set of data to provide a better interpretation of information [6,19]. In essence, it is a derivative of the orthogonal signal correction technique and distinguishes itself from PLS by eliminating systematic variation in X, which is orthogonal to Y [20]. This method is known to provide an improved model, facilitating better interpretability and understanding of the study design [21].

In PLS, the accuracy of the model is interpreted using R^2^ values, while the precision was expressed by RMSEC, RMSECV, and RMSEP values [8,22]. When it comes to OPLS models, R^2^Y indicates the goodness of fit for the components, while Q^2^ values interpret the ability of the prediction. As stated by Easmin et al. [6], RMSEE values indicate the average deviation and RMSECV values measure the predictive quality. Overall, the models with high regression coefficient values and low error values are identified as more accurate and precise [6,8].

Both PLS and OPLS regression analyses were adopted for developing statistical models in this study. Initially, PLS regression analysis was run to correlate different spectral regions with the Alpha-glu inhibitory activity of the crude extracts. In the next step, OPLS regression analysis was performed for the spectral regions that produced the best model under PLS analysis. In OPLS analysis, the processing of the FTIR data using filters was found to improve the accuracy and predictive ability of the models (Table 4). This approach was previously adopted by Easmin et al. [6] for FTIR spectral data analysis to build a rapid method for the detection of Alpha-glu inhibitory activity of *Phaleria macrocarpa*. According to their findings, the compounds consisting of C-H stretching of a methyl group, C=O stretching in ketone, aldehyde, and carboxylic acid groups, C-O stretching of an alcoholic group, C-H bending (out of plane) of aromatics and alkenes, and O-H stretching in phenolic and alcoholic groups were identified as the contributors for the Alpha-glu inhibition activity of *Phaleria macrocarpa*.

In a separate study, Saleh et al. [9] attempted to develop a validation model to correlate the Alpha-glu inhibitory activity of Salak fruit extracts with the FTIR data. According to their study, the C–H, C=O, C–N, N–H, and C–O vibrations representing esters, amines, and aldehydes were found to be responsible for the anti-Alpha-glu activity of Salak fruit extracts. Subsequently, Umar et al. [10] also employed PLS analysis to assist the untargeted metabolomics analysis of some *Curculigo* species. According to their study, the molecules representing the vibrational peaks of C-O stretching (trans-disubstituent), C-H bending (alkenes), and C-C bending (cyclohexane) might have contributed to the Alpha-glu inhibitory activity of a 70% ethanol extract of *Curculigo*. In addition, it was reported that the FTIR spectral regions that represent the functional groups of C=C, –OH, and –COOH were more likely to be linked with the Alpha-glu inhibitory activity of the *Curculigo* species.

## 5. Conclusions

This study explored the effectiveness of FTIR chemical mapping coupled with PLS regression and OPLS regression analyses in developing a rapid method for the detection of Alpha-glu inhibitory activity of CTF sub-fractions. Alpha-glu inhibitory activities of CTF sub-fractions were found to vary based on the cultivar difference and type of solvent used for the sequential extraction. The FTIR chemical mapping indicated the presence of a range of biomolecules and their distribution in different CTF sub-fractions. The PLS models rendered a strong correlation between some spectral regions and the inhibition of Alpha-glu enzymes, where the spectral regions were attributed to the phenolic and alcoholic compounds of CTF extracts. OPLS models also rendered a strong correlation between the data and provided a better interpretation of the model parameters. Therefore, PLS and OPLS methods can be used as effective multivariate tools to develop models to detect the Alpha-glu enzyme inhibitory potential of biomaterials. Nonetheless, additional research in this domain is warranted to offer a deeper understanding of the applicability of this technique to potential nutraceuticals and pharmaceuticals.

## Figures and Tables

**Table 1:** The half-maximal inhibitory concentration of CTF crude extracts for the inhibition of Alpha-glu enzyme activity.

Cultivar	IC_50_ Value/ppm		
	Hexane	EtOAc	MeOH
COM	18.84 ± 0.41 ^b(A)^	56.64 ± 3.37 ^b(A)^	403.32 ± 17.24 ^b(B)^
GT	8.38 ± 0.52 ^a(A)^	8.56 ± 0.30 ^a(A)^	22.53 ± 0.26 ^a(B)^
RT	40.12 ± 1.76 ^c(B)^	9.78 ± 0.73 ^a(A)^	70.43 ± 6.31 ^a(C)^
SR	61.18 ± 2.21 ^d(C)^	12.58 ± 0.37 ^a(A)^	25.39 ± 1.05 ^a(B)^
TT	65.91 ± 3.92 ^d(C)^	7.82 ± 0.40 ^a(A)^	36.51 ± 0.84 ^a(B)^

Each value in the table represents the mean of three replicates ± standard deviation. The means that do not share a similar simple superscript letter within the same columns (IC_50_ for the inhibition of Alpha-glu enzyme activity of different cultivars belong to same solvent extract type) are significantly different at 95% confidence (α = 0.05), while the means that do not share a similar capital superscript letter within the same rows (IC_50_ for the inhibition of Alpha-glu enzyme activity of different solvent extract types of same cultivar) are significantly different at 95% confidence (α = 0.05). Abbreviations: IC_50_, half-maximal inhibitory concentration.

**Table 2:** FTIR spectral wavenumbers of bands corresponding to different sub-fractions of CTF.

Mode of Vibration	Hexane Wavenumber Range * (cm^−1^)	EtOAc Wavenumber Range * (cm^−1^)	MeOH Wavenumber Range * (cm^−1^)	Functional Group
O-H stretching, H-bonded, normal polymeric OH stretch	nd	3442–3470	3345–3376	Alcohols, Phenols
C-H (CH_2_) asymmetric stretching	2924–2925	2924–2925	2925–2927	Alkanes
C-H (CH_2_) symmetrical stretching	2855–2856	2855–2856	2855–2857	Alkanes
C=O carbonyl ester stretching	1745	1744–1745	1742–1744	Esters
C=O stretching	nd	1628–1642	nd	Aromatic-C(O)-OH.
C=C-C stretch	nd	nd	1604–1611	Aromatic rings
C=O stretch (amide I)	nd	nd	1604–1611	Amides
C=C-C stretch	nd	nd	1523–1525	Aromatic rings
Amide II	nd	nd	1523–1525	Nitrogen compounds
C-H bending (CH_2_, CH_3_)	1462–1463	1462–1463	-	Alkanes
C=C stretch	nd	nd	1448–1451	Aromatic rings
O-H bending	nd	nd	1411	Phenol or tertiary alcohols
C-H bending (CH_3_)	1374–1376	1374–1375	-	Alkanes
C-O stretching	nd	nd	1267–1284	Phenols
	1231–1235	1230–1232	nd	Ester linkages
C-O stretching	1161–1169	1161–1165	nd	Ester linkages
	1108–1111	1109–1110	1106–1107	Secondary alcohols, Ester linkages
C-O stretching	nd	nd	1052–1055	Alcohols
=C-H bend	nd	nd	996–999	Alkenes
C-C vibration	nd	nd	926–927	Alkanes
(CH_2_)_n_ bending	723	722–723	nd	Hydrocarbons
O-H out-of-plane bend	nd	nd	593–627	Alcohols

* Range of variation in wavenumbers at a particular peak among the different coconut cultivars, namely, Gon Thembili, Ran Thembili, San Raman, Tall × Tall, Commercial hybrid. Abbreviation: nd, peak not detected.

**Table 3:** Statistical parameters of PLS models developed for Alpha-glu inhibitory activity and FTIR spectral data of crude extracts of CTF.

Model No	Region	Rc^2^	RMSEC	Rcv^2^	RMSECV	Rp^2^	RMSEP
1	A	0.95	23.41	0.89	34.97	0.89	31.95
2	B	0.59	64.83	0.33	84.50	0.50	62.81
3	C	0.90	32.42	0.81	45.61	8.80	39.01
4	D	0.89	33.52	0.85	40.04	0.88	30.09
5	E	0.96	19.34	0.93	26.58	0.98	13.91
6	C,D,E	0.97	16.88	0.94	24.48	0.97	15.80
7	D,E	0.96	21.16	0.92	28.82	0.95	20.06
8	A,C,D,E	0.97	18.17	0.94	26.23	0.96	16.70
9	A,D,E	0.97	17.31	0.94	25.31	0.97	16.51
10	A,E	0.96	20.25	0.92	28.99	0.91	26.18
11	A,C,E	0.97	18.20	0.93	27.50	0.93	23.14
12	A,B,C,D,E	0.98	13.23	0.96	21.78	0.97	14.74

A: 3700–2800 cm^−1^, B: 1800–1700 cm^−1^, C: 1700–1500 cm^−1^, D: 1500–900 cm^−1^, E: 900–500 cm^−1^. Rc^2^, coefficient of determination of calibration; Rp^2^, coefficient of determination of prediction; Rcv^2^, coefficient of determination of cross-validation; RMSEC, root mean square errors of calibration; RMSEP, root mean square errors of prediction; RMSECV, root mean square errors of cross-validation.

**Table 4:** Statistical parameters of OPLS models developed for Alpha-glu inhibitory activity and FTIR spectral data of crude extracts of CTF.

Model No	Spectral Filter	R^2^Y	Q^2^	RMSEE	RMSECV
I	Normal	0.933	0.916	26.430	27.997
II	1st derivative	0.975	0.965	16.301	18.115
III	2nd derivative	0.980	0.969	14.246	16.936
IV	3rd derivative	0.983	0.976	13.017	15.120
V	MSC	0.978	0.960	15.121	19.371
VI	SNV	0.977	0.961	15.559	19.132

Abbreviations: MSC, multiplicative signal correction; SNV, standard normal variant; R^2^Y, regression coefficient; Q^2^, predictive regression coefficient; RMSEE, root mean square error of estimation; RMSECV, root mean square error of cross-validation.

## Data Availability

The original contributions presented in this study are included in the article. Further inquiries can be directed to the corresponding authors.
